# Psychometric Properties of the Participation Scale among Former Buruli Ulcer Patients in Ghana and Benin

**DOI:** 10.1371/journal.pntd.0003254

**Published:** 2014-11-13

**Authors:** Janine de Zeeuw, Marlies Douwstra, Till F. Omansen, Ghislain E. Sopoh, Christian Johnson, Richard O. Phillips, Marike Alferink, Paul Saunderson, Tjip S. Van der Werf, Pieter U. Dijkstra, Ymkje Stienstra

**Affiliations:** 1 University of Groningen, University Medical Center Groningen, Department of Internal Medicine/Infectious Diseases, Groningen, The Netherlands; 2 Programme National de Lutte contre la Lèpre et L'Ulcère de Buruli, Ministère de la Santé, Cotonou, République du Bénin; 3 Fondation Raoul Follereau, Cotonou, République du Bénin; 4 Kwame Nkrumah University of Science and Technology, School of Medical Sciences, Department of Medicine, Kumasi, Ghana; 5 University of Groningen, University Medical Center Groningen, Department of Health Sciences, Groningen, The Netherlands; 6 American Leprosy Missions, Greenville, South Carolina, United States of America; 7 University of Groningen, University Medical Center Groningen, Department of Rehabilitation and Department of Oral and Maxillofacial Surgery, Groningen, The Netherlands; University of Tennessee, United States of America

## Abstract

**Background:**

Buruli ulcer is a stigmatising disease treated with antibiotics and wound care, and sometimes surgical intervention is necessary. Permanent limitations in daily activities are a common long term consequence. It is unknown to what extent patients perceive problems in participation in social activities. The psychometric properties of the Participation Scale used in other disabling diseases, such as leprosy, was assessed for use in former Buruli ulcer patients.

**Methods:**

Former Buruli ulcer patients in Ghana and Benin, their relatives, and healthy community controls were interviewed using the Participation Scale, Buruli Ulcer Functional Limitation Score, and the Explanatory Model Interview Catalogue to measure stigma. The Participation Scale was tested for the following psychometric properties: discrimination, floor and ceiling effects, internal consistency, inter-item correlation, item-total correlation and construct validity.

**Results:**

In total 386 participants (143 former Buruli ulcer patients with their relatives (137) and 106 community controls) were included in the study. The Participation Scale displayed good discrimination between former Buruli ulcer patients and healthy community controls. No floor and ceiling effects were found. Internal consistency (Cronbach's alpha) was 0.88. In Ghana, mean inter-item correlation of 0.29 and item-total correlations ranging from 0.10 to 0.69 were found while in Benin, a mean inter-item correlation of 0.28 was reported with item-total correlations ranging from −0.08 to 0.79. With respect to construct validity, 4 out of 6 hypotheses were not rejected, though correlations between various constructs differed between countries.

**Conclusion:**

The results indicate the Participation Scale has acceptable psychometric properties and can be used for Buruli ulcer patients in Ghana and Benin. Future studies can use this Participation Scale to evaluate the long term restrictions in participation in daily social activities of former BU patients.

## Author Summary

Buruli ulcer is a stigmatising condition caused by infection with *Mycobacterium ulcerans*. Besides the long term medical consequences, Buruli ulcer may lead to participation restrictions in social life. The Participation Scale intends to assess perceived participation restrictions; however, this instrument has been developed in patients affected by leprosy and other disabling conditions, and has never been used before among Buruli ulcer patients. We aimed to analyze the reliability and validity of the Participation Scale among former Buruli ulcer patients in Ghana and Benin. This study included former Buruli ulcer patients from 2 different treatment sites, along with their relatives and healthy community controls residing in similar geographical areas. Former Buruli ulcer patients were interviewed using the Participation Scale, Buruli Ulcer Functional Limitation Score, and the Explanatory Model Interview Catalogue to measure stigma. Relatives and healthy community controls were interviewed using the Participation Scale. We tested the Participation Scale for discrimination, floor and ceiling effects, internal consistency, inter-item correlation, item-total correlation and construct validity. The results of the analysis suggest that the Participation Scale has acceptable psychometric properties. As such, the instrument can be used to assess participation restrictions among former Buruli ulcer patients in Ghana and Benin.

## Introduction

Buruli ulcer (BU) is a neglected tropical disease caused by *Mycobacterium ulcerans* (MU) and has been reported in more than 30 countries predominantly in tropical or subtropical areas [1]. Most burdened region is West Africa of which Côte d'Ivoire, Ghana and Benin have reported large number of cases in 2012 [2]. BU presents as a small nodule or a plaque sometimes accompanied with edema. Later, the lesion breaks open, and a characteristic lesion presenting with undermined edges appears [3]. BU affects skin, subcutaneous tissue, muscles and sometimes bone resulting in scarring, deformities, and contractures; sometimes, amputation is necessary [4], [5]. This in turn may lead to long-term physical disability such as restriction in movement of joints [6] and functional limitation [7]. Apart from these problems, stigmatisation due to superstitious beliefs or inappropriate or delayed treatment may lead to considerable impact on functioning in social life [8], [9], such as change in occupation, unemployment, school dropout, and economic burden [10], [11].

The World Health Organization's (WHO) model of International Classification of Functioning, Disability and Health (ICF) [12] describes disabilities in terms of impairments, activity limitations and participation restrictions. Participation restrictions are being viewed in this model through a social perspective and are defined as; ‘any problem an individual may experience in involvement in life situations’ [12]. Visible signs, activity problems and stigma are associated with an increased risk for participation restrictions [13], [14]. Various instruments have been developed to measure participation restrictions for populations in affluent and industrialized countries. The Participation Scale (P-scale) designed to measure the severity of participation restrictions as perceived among persons with disabilities, is the only questionnaire developed for the context of low and middle-income countries [15]. The P-scale has been applied to study the psychometric properties among persons with leprosy, poliomyelitis and spinal cord injuries aged at least 15 years [15]. The instrument is considered culturally free, generic, easy to administer by local staff, and has been recommended for patients with BU [15], however properties in such patients have not yet been tested. Therefore, the aim of this study was to test the psychometric properties of the P-scale among former BU patients in Ghana and Benin.

## Methods

### Study population

Former patients with BU were identified using medical records kept by the Agogo Presbyterian Hospital in Ghana and the Centre de Dépistage et de Traitement de l'Ulcère de Buruli in Lalo, Benin. These patients were clinically diagnosed as BU patients in accordance with the WHO case definition [16]. Participants had to be at least 15 years; with treatment for BU between 2005–2011 to be completed at least 3 months before the start of the current study. We also included at least 50 healthy community controls in each of the two countries to test the discriminative potential of the P-scale. To strive for an equal distribution in patients and controls regarding location, age and sex, healthy community controls were recruited from villages located in the study area; we attempted to have these control participants match with our former patients for age (+5/−5 years) and sex. Finally, former patients with BU were asked to help identify a relative to be approached who could be included in the study to act as representative to be able to test one of the hypothesis for the construct validity of the P-scale.

### Questionnaires

#### Participation restrictions

The P-scale contains 18 items covering eight of the nine major life domains defined by the ICF; learning and applying knowledge (1 item), communication (1 item), mobility (3 items), self-care (1 item), domestic life (2 items), interpersonal interactions and relationships (4 items), major life areas (3 items) and community, social and civic life (3 items). The major life domain “general tasks and demands” is not asked for in the P-scale. For the majority of the items, the participant is asked for comparison with a ‘peer’. A peer is described as ‘someone who is similar in all socio-cultural, economic and demographic aspects, except for the disease or disability’. Each item is subdivided into an objective and subjective part. The objective response options are yes, no, sometimes, not specified, not answered, or irrelevant. If the response is ‘no’ or ‘sometimes’ the participant is questioned to rate the problem for which response options are scored on a 4 point Likert-scale; 1 is no problem, 2 is small problem, 3 is medium problem, and 5 is large problem. The total score is the sum of all item scores and a higher score indicates more participation restrictions. The P-scale was developed and validated among people with disability related conditions or other stigmatising conditions in Brazil, Nepal and India [15]. That study showed internal consistency of 0.92, intra-tester stability of 0.83, inter-tester reliability of 0.80, both evaluated using Intra-Class Correlation coefficient [15] and test-retest reliability using Intra-Class Correlation_agreement_ of 0.90 [17]. Other studies found internal consistency varying from 0.87 to 0.93 in India and Nepal [13], [17], [18]. Different factor models have been found, a one-factor model, labeled as participation [15] and a two-factor model, assessing work-related participation and general participation [17].

#### Functional limitations

The Buruli Ulcer Functional Limitation Score (BUFLS) previously developed [19] and validated [20] for patients with BU was used to assess functional limitations among former BU patients. Questions aim to gain insight in the study participants' perception on abilities in 19 daily tasks divided in food preparation, personal care, daily work activities and mobility. The BUFLS uses an ordinal response scale; 0 points reflect an activity without problems, 1 point indicates an activity is performed with difficulties and 2 points imply an activity is not possible. The total score is the sum score divided by the maximal score applicable for the patient and multiplied by 100. A higher score indicates more functional limitations. The BUFLS has been validated among former BU patients in Ghana and Benin in the local languages (Twi and Fon). The validity of the BUFLS as established by correlations with range of motion and the global impression of functional limitation is acceptable [20]. The internal consistency established with Cronbach's alpha is 0.82 and the inter-observer reliability as measured with the intra-class correlation coefficient is 0.86. The mean difference of 4.0 between test-retest results implies that the BUFLS is not suitable for longitudinal individual assessment [20].

#### Perceived stigma

To assess the level of perceived stigma the Explanatory Model Interview Catalogue (EMIC) was used [21]. The EMIC contains a subset of questions aiming to measure perceived stigma [22] of which 15 items have been previously administered to patients with BU in Ghana [8] and Benin [23]. Each question contributes equally and the response options are 3 points for yes, 2 points for possibly and 1 for uncertain and 0 for no. In this study, sensitive questions on sexual behavior and fertility were not asked to patients younger than 20 years of age. To compensate for items not asked, the total stigma score was scored as the percentage of the maximum score a participant could receive on the questions applicable for that participant. Higher scores indicate higher levels of perceived stigma. In Ghana an internal consistency of 0.64 has been reported [8] whereas the version of the EMIC as used previously in Benin was not tested for its internal consistency [23].

### Translation

The English P-scale (version 6.0) was translated into Twi (one of the local languages in Ghana) and French (Benin) and back translated. To ensure correct translation into the local language in Benin, Fon, an interview conducted by a native speaker was recorded and back translated by another native speaker without knowledge of the initial French version of the P-scale. Thereafter the translated, back translated and initial versions were compared. Each item was checked for the correct words used that were appropriate for the target group and whether the meaning of the question was maintained. Both in Ghana and Benin, pilot studies were conducted to study understandability of the wording, the question and the response options. Examples not suitable for the West African context, like bazaars, or tea/coffee shops were removed.

### Procedures

In both countries, face validity of the P-scale was established among medical doctors, nurses, a physical therapist, a social scientist and a BU coordinator. The conceptualization of the construct participation restriction, the relevance and importance of each item, the understandability of the peer concept, and the response option for the target population was discussed. In both countries, two native language speaking interviewers participated in a training on the objective of the interview, and conducting the interviews in accordance with the available manuals, the P-scale Users Manual (version 6.0) and BUFLS manual (2012) to prevent biases during interviewing. The P-scale was adapted for relatives. Each question was adapted in such a way that the relative could answer for the former BU patient. For example the first question of the P-scale was changed into;

Does he/she have equal opportunity as his/her peers to find work?

How big is that problem to him/her?

### Discrimination

To establish discrimination, it was determined a significant difference (α value of 0.05) in P-scale sum scores between former patients with BU and healthy controls indicates the instrument has discriminative value.

### Floor and ceiling effects

Floor and ceiling effects were determined to be present when ≥15% of the respondents scored the lowest (0) or the highest (90) possible score on the P-scale.

### Internal consistency

A Cronbach's alpha was calculated to determine internal consistency. A value of ≥0.70 was considered sufficient [24].

### Construct validity

To determine construct validity, hypotheses were formulated *a priori* accounting for both countries together. To positively rate construct validity ≥75% (5 out of the 6) of the a priori hypothesis need to be confirmed [24].

The hypotheses formulated are:

Hypothesis 1: Former patients with BU with visible deformities have significantly higher P-scale scores than those without.
Hypothesis 2: Former patients with BU with a joint involved have significantly higher P-scale scores than those without.
Hypothesis 3: Former patients with BU who have changed their profession due to BU have significantly higher P-scale scores than those who continued the same job.
Hypothesis 4: A positive correlation (0.4–0.8) exists between the P-scale sum scores and the BUFLS sum scores.
Hypothesis 5: A positive correlation (0.4–0.8) exists between the P-scale sum scores and sum stigma scores.
Hypothesis 6: A positive correlation (0.4–0.8) exists between the P-scale sum scores of former patients with BU and their relatives.

### Ethical consent

The Medical Ethical Review Committees of the Kwame Nkrumah University of Science and Technology; School of Medical Sciences, Komfo Anokye Teaching Hospital in Ghana (ref: CHRPE/RC/127/12) and Ministry of Health in Benin (ref: N^0^1961/MS/DC/SGM/DRF/SRAO/SA) approved the study. Before the interview started, the aim of the interview was explained and written informed consent was obtained from the participants. The participants were informed on the voluntary participation and confidentiality of the study.

### Statistics

Data were analyzed using Statistical Package for the Social Sciences (SPSS) 20.0. To correct for missing values of P-scale questions of former BU patients, imputation using individual mean subscale scores was used. In case a missing value represented a 1-item subscale, the total mean score was calculated to fill out the missing value. Non-parametric analysis was computed as all questionnaire data was positively skewed. The Mann-Whitney U test or Chi-square was used to analyze possible differences in clinical signs, socio-economic, and demographic factors between countries and P-scale scores. To control for differences across countries, analysis for Ghana and Benin were performed separately. When striking differences between countries were not found analysis was performed taking both countries together. Cronbach's alpha, inter-item correlation and item-total correlation were used to analyze internal consistency. Spearman's rho correlations and confidence intervals were used to test the associations between the P-scale and the scores of the BUFLS, EMIC and P-scale administered among relatives. Calculation of confidence intervals into z-scores allowed interpretation as effect sizes [25] to study differences across countries. An effect size of 0.10 is considered small with a negligible practical importance, an effect size of 0.30 is considered medium with a moderate practical importance, and an effect size of 0.50 is considered large and of crucial importance [25]. Mann-Whitney U test was computed to analyze differences in P-scale scores between former patients with BU and healthy controls.

## Results

A total of 143 former patients with BU, with 137 relatives, and 106 healthy community controls were included in the study. Table 1 depicts basic characteristics of the former patients with BU and health community controls across countries and the P-scale scores. Four former patients with BU required imputation of missing values based on sum scores. Analysis of differences in basic characteristics between countries showed significant differences in type of treatment and visible deformities. In Ghana, differences between sex on P-scale sum scores were not found while in Benin, women reported significantly (*P = *.001) higher P-scale sum scores (median 23, IQR; 9;40) compared to men (median 7, IQR; 3;17). For both countries taken together, P-scale sum scores were significantly higher (*P = *.001) for women (median 19, IQR; 7;33) compared to men (median 9, IQR; 3;24).

**Table 1:** Baseline characteristics of former BU patients (*n* = 143) and controls (*n* = 106).

		Former BU patients		Healthy community controls	
Variables		Ghana (*n* = 75)	Benin (*n* = 68)	Ghana (*n* = 50)	Benin (*n* = 56)
Age at time of inclusion, Median (IQR)		27 (19;33)	25 (18;43)	28.5 (21;37)	31 (24;41)
Sex, male (%)		33(44)	31(46)	23(46)	31(55)
Type of treatment**	Antibiotics, n (%)	55 (73)	29 (43)	-	-
	Antibiotics & surgery, n (%)	20 (27)	39 (57)	-	-
Nr. of lesions	1, n (%)	68 (91)	61 (91)	-	-
	>1, n (%)	7 (9)	6 (9)	-	-
Location of lesion*	Upper limb, n (%)	35 (45)	18(27)	-	-
	Lower limb, n (%)	35 (45)	45 (68)	-	-
	Trunk/head/neck, n (%)	8 (10)	3 (5)	-	-
Joint involved, yes (%)		24 (36)	27 (42)	-	-
Visible deformity, yes (%)**		1 (2)	17 (32)	0	0
Profession	Employed, n (%)	59 (79)	45 (66)	41(82)	52 (95)‡
	Unemployed, n (%)	1 (1)	8 (12)	1 (2)	0
	Student, n (%)	15 (20)	15 (22)	8 (16)	3 (5)
Change occupation, yes (%)		6 (9)	2 (3)	-	-
Literacy, yes (%)		19 (26)	17 (42)	-	-
Participation restriction score (Median, IQR, range)		13 (5;29), 0–78	13 (4;30), 0–72	2 (0.3;8), 0–49	1 (0;3), 0–23

*Ghana: 1 missing and 4 patients with more lesions included, Benin: 4 missing and 2 patients with more lesions,

**significant difference between Ghana and Benin, ‡ 1 missing.

### Discrimination

Age and sex did not differ significantly between former patients with BU and healthy controls (Table 1). P-scale scores among former patients with BU (median 13, IQR; 4;29) were significantly higher (*P*<.001) than that of healthy controls (median 2, IQR; 0;6). Similar results were found when looking at discriminative value of the P-scale in both countries.

### Floor and ceiling effects

Overall, no floor and ceiling effects were found as 6% scored the lowest possible sum score (0) and none of the participants scored the highest possible (90) sum score.

### Internal consistency

The median scores of the separate items according to the different subscales of the P-scale across countries are shown in Table 2. For Ghana the mean inter-item correlation was 0.29 (range −0.13–0.71) while for Benin this was 0.28 (range −0.22–0.95). In Ghana, lowest item-total correlation (0.10) was found for the item ‘confident to try to learn new things’ of and highest item-total correlation (0.69) was found with ‘work hard as your peers do’. In Benin, lowest item-total correlation (−0.08) was found with ‘comfortable meeting new people’ and highest (0.79) was found with ‘mobility house/village as other people’. The 18 item P-scale had a Cronbach's alpha of 0.88 (124 participants). A Cronbach's alpha of 0.88 was found among participants in Ghana (n = 57) and was similar (0.87) in Benin (n = 67).

**Table 2:** Percentages scoring not restricted, no, small, medium and large problem per question of the P-scale.

Domain of participation restriction	Question	Ghana, %					Benin, %				
		Not restricted	No problem	Small problem	Medium problem	Large problem	Not restricted	No problem	Small problem	Medium problem	Large problem
Major life areas	Work opportunity	31	8.5	2.8	9.9	47.9	45.6	4.4	1.5	2.9	45.6
	Work as hard	41.3	5.3	5.3	13.3	34.7	41.8	4.5	-	4.5	49.3
	Contribute economically	33.8	8.5	2.8	12.7	42.3	38.8	35.8	-	7.5	17.9
Mobility	Visits outside village	77.3	-	1.3	5.3	16	55.2	14.9	1.5	10.4	17.9
	Mobility house/village	88	1.3	2.7	2.7	5.3	75	5.9	-	4.4	14.7
	Visit public places	84	1.3	1.3	2.7	10.7	76.1	1.5	-	7.5	14.9
Community, social and civic life	Take part in festivals and rituals	80	4	-	2.7	13.3	61.8	19.1	-	8.8	10.3
	Take part in recreational and social activities	74	5.5	6.8	2.7	11	58.8	13.2	-	11.8	16.2
	Socially active	72.9	5.7	1.4	2.9	17.1	36.4	30.3	-	4.5	28.8
Interpersonal interactions and relationships	Respect in community	73.3	2.7	2.7	6.7	14.7	80	1.5	-	6.2	12.3
	Long-term relationship	70.8	1.4	1.4	2.8	23.6	88.1	1.5	-	-	10.4
	Visits in the community	85.1	1.4	-	6.8	6.8	80.6	-	-	10.4	9
	Meeting new people	64	2.7	-	2.7	30.7	97	-	-	1.5	1.5
Self-care	Take care of self	83.6	-	1.4	4.1	11	95.6	-	-	-	4.4
Domestic life	Household work	84	6.7	1.3	1.3	6.7	81.8	7.6	-	1.5	9.1
	Helping other people	82.4	2.7	2.7	5.4	6.8	60.3	11.8	-	20.6	7.4
Communication	Opinion in family discussion	69.7	6.1	3	4.5	16.7	47.7	30.8	1.5	10.8	9.2
Learning and applying knowledge	Confident to learn new things	86.7	4	-	4	5.3	91	-	-	1.5	7.5

### Construct validity

Hypothesis 1: P-scale scores of former patients with BU with visible deformities (n = 18, median 19, IQR; 11; 45) were significantly (*P* = .023) higher than those without visible deformities (n = 84, median 8.5, IQR; 3; 25). (Hypothesis accepted).

Hypothesis 2: P-scale scores of former patients with BU with joint involvement (n = 51, median 18, IQR; 8; 32) were nearly significantly (*P* = .056) higher than those without joint involvement (n = 80, median 9.5, IQR; 3; 27). (Hypothesis rejected).

Hypothesis 3: P-scale scores of former patients with BU that changed occupation were higher (n = 8, median 16.5, IQR; 5; 38) compared to those that continued the same occupation (n = 117, median 13, IQR; 5; 29) but this difference did not reach statistical significance (*P* = .809). (Hypothesis rejected).

Hypothesis 4: A Spearman's rank correlation of 0.67 was found between the BUFLS and P-scale scores. (Hypothesis accepted).

Hypothesis 5: A Spearman's rank correlation of 0.53 was found between the EMIC and the P-scale. (Hypothesis accepted).

Hypothesis 6: A Spearman's rank correlation of 0.80 was found between the P-scale sum scores of former BU patients and relatives. (Hypothesis accepted).

The associations between functional limitations, perceived stigma and participation restrictions separately for Ghana and Benin are depicted in Table 3. A small effect size [25] was found with respect to functional limitation and participation restrictions implying that associations between constructs are almost similar across Ghana and Benin. When looking at the difference in association between perceived stigma and participation restrictions in both countries, a medium effect size appeared meaning that the difference in association between both countries is of moderate importance. Finally, the association between the participation score as answered by a relative of the patient and the patient differed largely between Ghana and Benin. In Ghana a Spearman's rank correlation of 0.70 was found between the P-scale sum scores of former BU patients and relatives, while in Benin a Spearman's rank correlation of 0.92 was found between the P-scale sum scores of former BU patients and relatives.

**Table 3:** Spearman's rank correlations, 95% confidence intervals and effect sizes for differences in correlations between the P-scale scores of former BU patients and relatives, and with functional limitations and stigma.

	P-scale Ghana	P-scale Benin	Effect sizes for differences in correlations
BUFLS: functional limitations	0.64(0.48;0.76)	0.71(0.57;0.81)	0.13
EMIC: perceived stigma	0.64(0.48;0.76)	0.36(0.13;0.55)	0.38
Relative: participation restrictions	0.70(0.56;0.80)	0.92(0.87;0.95)	0.72

## Discussion

This study aimed to test the psychometric properties of the P-scale among former patients with BU in Ghana and Benin. The findings indicate acceptable psychometric properties for assessing participation restrictions in former patients with BU aged at least 15 years. Findings revealed good discriminative potential between former BU patients and controls, which are similar to those in the initial development study of the P-scale of van Brakel et al. (2006). Internal consistency was good and consistent with those found in previous studies [13], [17], [18]. Floor and ceiling effects were absent, as in earlier studies [13], [17].

Our study confirmed 4 out of the 6 hypotheses. Moderate to strong relations were found with functional limitation, stigma and participation problems indicated by relatives. Associations with functional limitation and stigma are corresponding with associations previously found [13], [14]. When looking at country level, similar associations between functional limitations and participation restrictions emerged. However correlations between perceived stigma and participation restrictions were moderately different, and the association between relatives and patients was strikingly different. It is possible that translation of the instrument and observer differences could have resulted in distinct associations in our study. However, in both countries, after translation and back-translation of the P-scale, the correct formulation of the content of the questions was discussed extensively. In addition, in Benin and Ghana a pilot study was conducted to ensure reliability between the trained native-speaking interviewers and interviews were reviewed regularly during data collection. However the equivalence of pervious scores and scores in this study should be compared and a factor analysis should be performed to assess measurement invariance which we initially planned, but the sample sizes of both groups (Ghana and Benin) were too small. It is also plausible that the experience of participation restrictions and stigma may be different in Ghana and Benin due to cultural differences, and that evaluation of relatives regarding the experience of participation restrictions for former patients with BU could have been interpreted differently due to differences in type of relatives in Ghana and Benin or local cultural differences. The findings of this study indicate the P-scale has acceptable psychometric properties and can be used for BU patients in Ghana and Benin, but the cross-cultural applicability of the instrument should deserve further study. Recently, it was also recommended that the P-scale should be studied more extensively on cultural validity in a new cultural context [26]. In addition future studies should focus on psychometric testing using pre-post scores to provide information regarding the responsiveness of the P-scale, test-retest reliability and intra- rater and inter-rater reliability. Since many of the BU patients are children, further research is needed in order to also develop a suitable instrument for children up to 15 years old measuring participation restrictions and explore the extent of participation problems among these children. Gathering knowledge on participation problems among BU patients is essential in order to be able to develop a suitable intervention.

## Supporting Information

Checklist S1 STROBE checklist. ** √  =  what is described in the manuscript, NA =  Not applicable, ‡ described in a twin paper: Persisting social participation restrictions among former Buruli ulcer patients in Ghana and Benin.
(DOC)
